# Through-Drop Imaging of Liquid–Solid Interfaces: From Contact Angle Variations Along the Droplet Perimeter to Mapping of Contact Angles Across a Surface

**DOI:** 10.1021/acs.langmuir.4c00414

**Published:** 2024-04-15

**Authors:** Arthur Vieira, Ville Jokinen, Sakari Lepikko, Robin H. A. Ras, Quan Zhou

**Affiliations:** † Department of Electrical Engineering and Automation, School of Electrical Engineering, Aalto University, Maarintie 8, 02150 Espoo, Finland; ‡ Department of Chemistry and Materials Science, School of Chemical Engineering, Aalto University, Tietotie 3, 02150 Espoo, Finland; § Department of Applied Physics, Aalto University, 02150 Espoo, Finland; ∥ Centre of Excellence in Life-Inspired Hybrid Materials (LIBER), Aalto University, 02150 Espoo, Finland

## Abstract

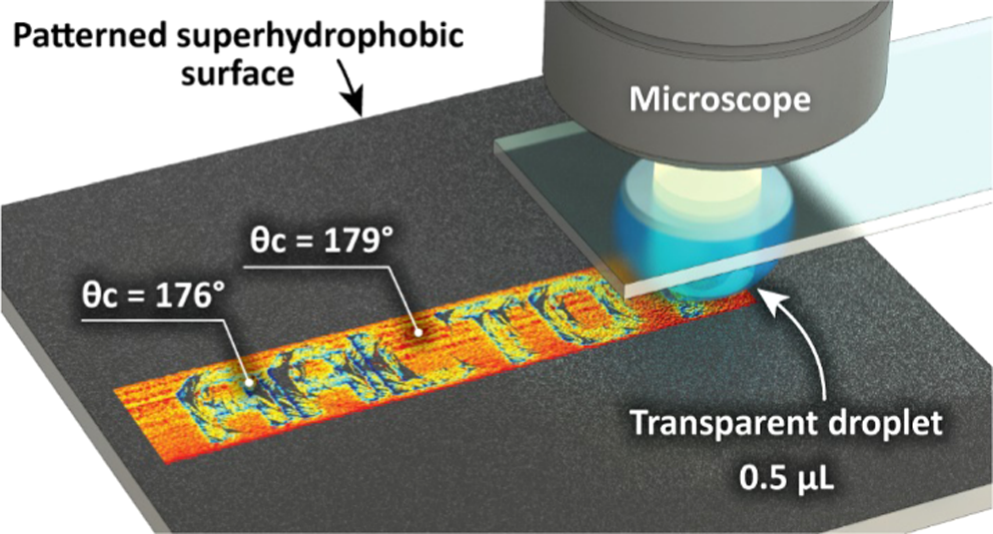

When a droplet interacts with a water-repellent surface, its triple-phase contact line typically exhibits varying contact angles, which can vary from point-to-point across the surface. Consequently, measuring the contact angles along the contact line would provide a better representation of the wetting properties of the surface than a single average contact angle. However, an effective method for estimating the local contact angle along the contact line on opaque hydrophobic surfaces is currently lacking. Here we present a method that combines through-drop imaging of the wetting interface during a sliding experiment with Finite Element Modeling of the droplet to estimate contact angle values along the contact line. Using this method, the mean advancing and receding contact angles were measured on four types of hydrophobic samples with contact angles between 99 and 178.9°. The method was further used to produce detailed advancing and receding contact angle maps of surfaces with wetting patterns with an unprecedented resolution of 3 μm.

## Introduction

A multitude of natural surfaces are water-repellent, such as lotus leaf,^1^ rose petals,^2^ butterfly wings,^3^ and bird feathers.^4^ Artificial surfaces with similar properties have been created with applications in fog-collection,^5^ self-cleaning,^6^ and micro- and nanoassembly.^7,8^ The performance of these surfaces depends on their wetting characteristics, which can vary spatially across their surface. In particular, the wetting state of a droplet is defined by the contact angle formed with such surfaces.^9^ Moreover, the state of the droplet is also dependent on the history of the interaction,^10^ leading to contact line (CL) shapes beyond the ideal circular shape and contact angle values that can vary along the CL at any given moment.^11^ For this reason, measuring the contact angles along the CL would better describe the wetting properties of such surfaces than a single average contact angle value.

Currently, there is no effective method for estimating the local contact angle along the CL on opaque hydrophobic surfaces. For hydrophilic samples the CL shape can be directly observed from the top-view,^12^ for example, using the tilting plate method where the sample is slowly tilted and gravity pulls the droplet downhill.^13^ Alternatively, the CL shape can be controlled to a prescribed shape.^14^ However, for hydrophobic surfaces, direct top-view observation is challenging because the CL is covered by the body of the droplet. Side-view contact angle goniometry is unable to accurately determine the shape of the CL and is limited to measuring the contact angle at only two points located on opposite sides of the droplet. In the superhydrophobic regime, the problem of an obscured CL is particularly noticeable, making measurements increasingly inaccurate at higher contact angle values.^15,16^ Although there are many techniques for measuring the CL shape in the hydrophobic regime, they either require specialized transparent surfaces, necessitate a stationary droplet during measurement, or cannot measure CL progression. Transparent samples can be imaged from underneath,^17,18^ using, e.g., inverted scanning laser confocal microscopy that can measure the three-dimensional (3D) shape of the droplet-sample-air system in real time.^19^ Alternatively, the CL shape can be estimated from the apparent diameter observed by rotating the side-view camera around the droplet-sample system,^20^ but the technique is limited to CLs with convex shape and the droplet must remain static during the measurement process. Many of these methods lack control over both the shape and the mobility of the droplet, complicating modeling of the interaction and subsequent contact angle estimation. For instance, in the tilting plate method, the droplet velocity is not controlled. Or, methods that use a micropipette to dispense and hold the liquid result in a droplet with variable and irregular shape. Recently, we demonstrated that a through-drop imaging method allows visualizing the shape of the wetting interface on opaque surfaces.^21^ The method can also estimate the mean contact angle with a precision down to 0.2°, where the results were verified using a Digital Holography Microscope (DHM) for contact angles above 178°. We also reported a method combining the through-drop imaging with force sensing that can separately measure the wetting forces due to surface tension from the forces due to the Laplace pressure.^22^ However, both methods assume the CL to be circular and do not estimate the local contact angles along the contact lines.

Here we report a method for estimating the contact angle values along the CL from the direct observation of the wetting interface during sliding experiments on hydrophobic and superhydrophobic samples. The method combines the CL shape obtained from through-drop images with precise droplet volume and position control. Based on this information, we calculate the shape of the droplet via Finite Element Modeling (FEM), from which the contact angles along the CL can be estimated. We measured average CL contact angle profiles on three types of superhydrophobic nanograss surfaces and a hydrophobic self-assembled monolayer surface, where the surface contact angle was found to vary by as little as 0.4° across the 1 × 0.35 mm^2^ measurement area. The segments of the CL exhibiting the advancing contact angle, *θ*_a_, receding contact angle, θ_r_, and values between them are also identified. Furthermore, we can generate θ_a_ and θ_r_ maps for patterned surfaces with an unprecedented spatial resolution of 3 μm, at the same scale as the surface features of ≤1 μm.

## Concept

**Figure 1:**
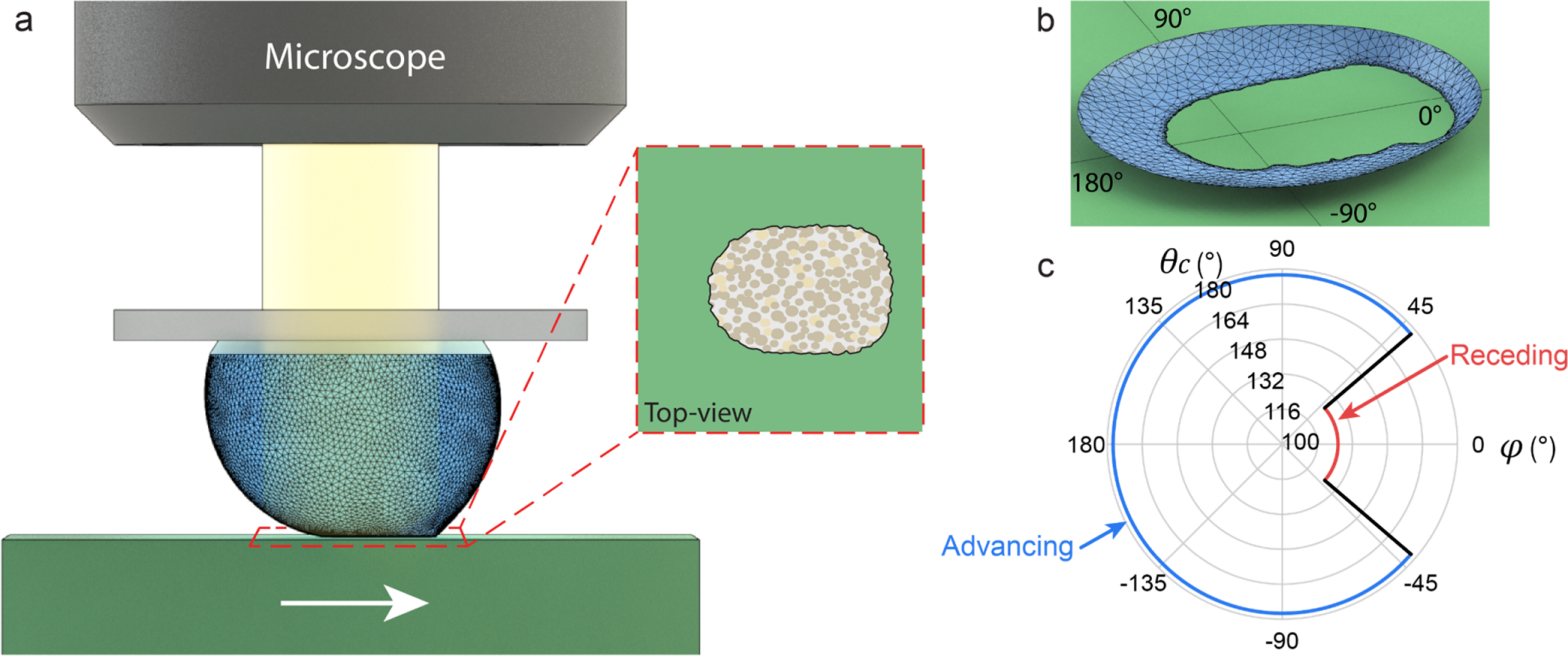
A transparent droplet probe allows measurements of the shape of the wetting interface. The contact angle values along the CL can be estimated from a FEM calculation of the shape of the droplet. (a) The transparent probe consists of a liquid droplet attached to a transparent glass slide. Coaxial illumination is used to image the wetting interface. The inset shows a top-view camera image of the interior of the droplet where the wetting interface appears bright due to the direct reflection of the imaging light. (b) A detailed droplet shape is produced by the FEM calculation (blue) based on the shape of the CL obtained from the top-view. A cross-section is shown, where the interior of the droplet is visible. (c) Illustration of contact angle values along the CL obtained from FEM, shown in polar coordinates. The blue line shows the section of the CL with advancing contact angle, and the red line shows the section of CL that has receding contact angle.

The method uses a water droplet probe attached to a transparent holding disk under a glass slide (Figure 1a). The sample is placed on a precision motorized XYZ stage. A top-view camera observes the wetting interface from above through the probe. For each measurement, the sample is pressed against the droplet and moved horizontally at a constant speed. A machine vision algorithm then analyses the top-view images to determine the shape of the wetting interface throughout the measurement (Figure 1a-inset).

For each top-view image frame, the state of the droplet is defined by the shape of the CL and the volume of the droplet. The sliding motion leads to an irregular CL shape (Figure 1a-inset and b) and induces both θ_a_ and θ_r_ along the CL, which arise due to contact angle hysteresis and chemical or topographical inhomogeneities. The contact angle can be plotted as a function of the azimuthal angle, φ, as shown in Figure 1c. This approach facilitates the characterization of θ_a_ and θ_r_ within a single measurement, encompassing the entire area traversed by the droplet during sliding. The shape of the droplet is calculated using FEM energy-minimization modeling, from which the value of the contact angle can be estimated for every point along the CL.

## Results

### Mapping Contact Angles Along the Contact Lines

**Figure 2:**
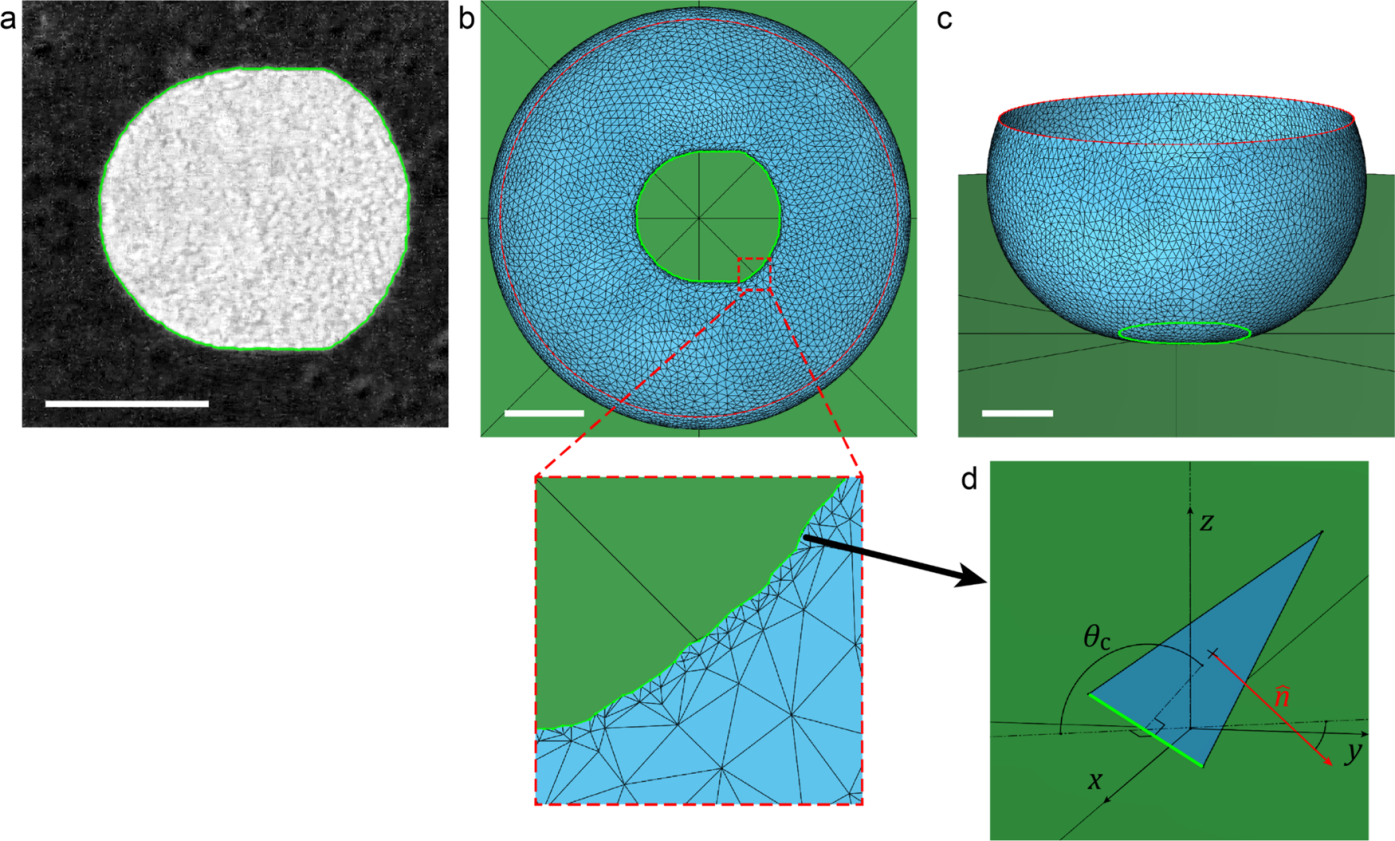
FEM calculation of the contact angles along the CL. (a) Example of a top-view image of the wetting interface on silicon nanograss (corrected with flat-field). The CL is identified with machine vision (green). (b) Calculation of the droplet shape, as seen from the top-view. The blue surface shows the water–air interface represented in the FEM method by a mesh of interconnected points. The inset shows a close-up of the mesh near the CL. (c) Bird’s eye view of the droplet. (d) Illustration of contact angle calculation. For every mesh facet belonging to CL, the contact angle is calculated as the angle between the surface normal of the facet and the *Z* axis. Scale bars: 200 μm.

Figure 2 shows how the contact angles are obtained from top-view images using FEM. In the top-view images, the wetting interface appears as a bright central region against a darker background shown in Figure 2a. Each top-view frame is analyzed to determine the CL, marked as a green outline in Figure 2. First, the image is corrected to remove shadows cast from imperfections present in the droplet-holding disk, based on flat-field correction method^23^ (See Materials and Methods section). A simple binary threshold method is used to find the shape of the wetting interface, from which the CL is taken as its perimeter.

After the shape of the CL is determined, the shape of the droplet is calculated using Surface Evolver,^24^ which is a FEM tool for calculating surfaces shaped by surface tension and other energies. For each calculation, CL acts as a boundary condition at the bottom of the droplet. At the top, the surface of the droplet is constrained to a circle with the same radius as the droplet-holding disk (red outline in Figure 2) (see Materials and Methods section and SI Section 3 for more information on how key droplet geometry parameters are determined). In the FEM calculation, the shape of the water–air surface of the droplet is represented by a mesh of triangles, as seen in the Figure 2b inset. After the shape of the droplet is found, the contact angle can be calculated for each CL segment (green) based on the geometry of the triangle (blue) to which the CL segment belongs, as seen in Figure 2d. The contact angle θ_c_ is calculated as the angle between the normal vector of the facet, *n̂* (red), and the Z axis, *ẑ*:
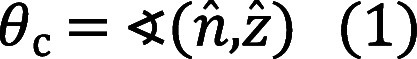
1The contact angle value is then associated with the *XY* coordinate of the center of the CL edge used in the calculation.

### Sliding Measurements

**Figure 3:**
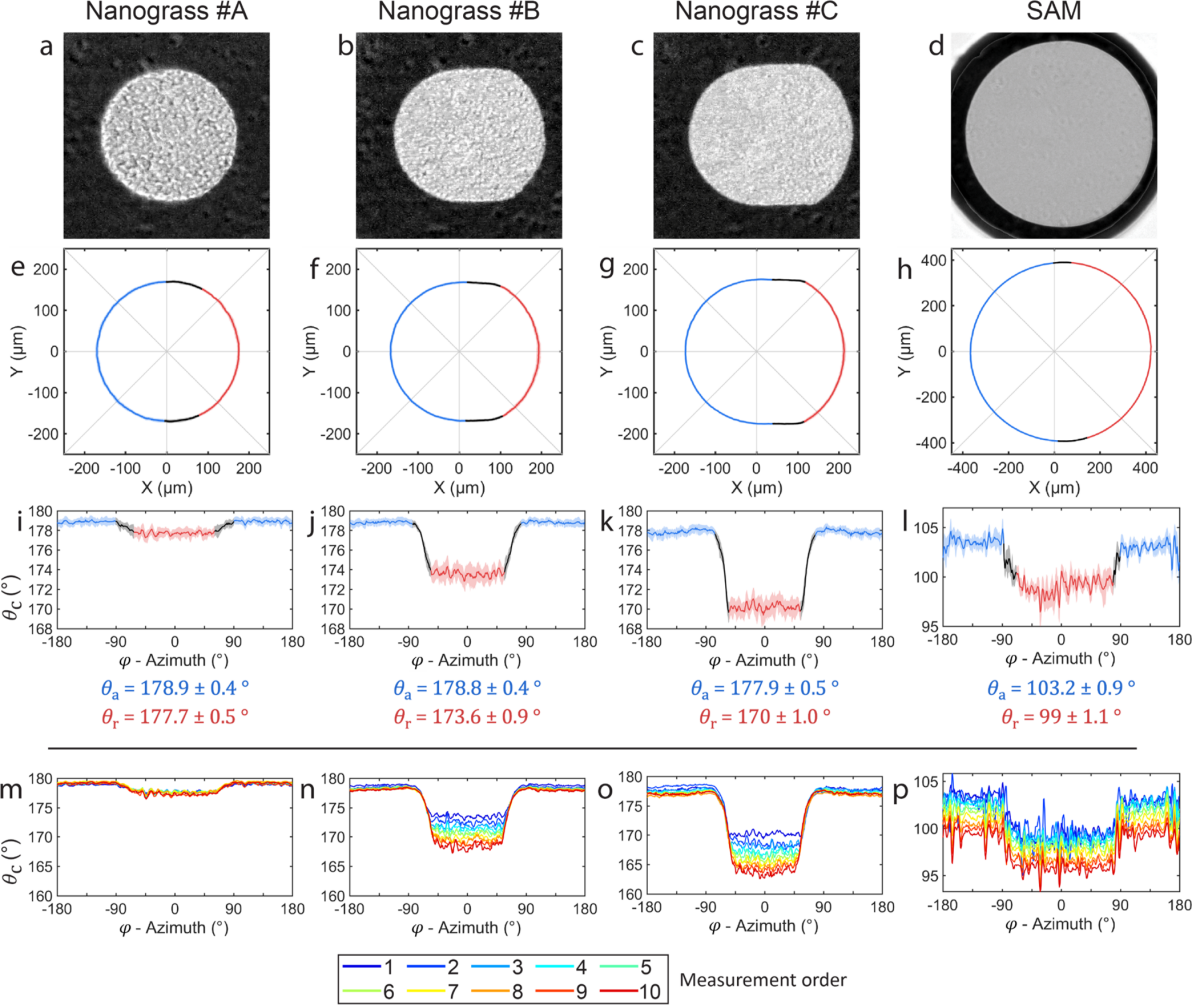
Sliding wetting interface shape and contact angle on three types of silicon nanograss, #A, #B, and #C, and one SAM sample. (a–d) Top-view image of the wetting interface of the different samples during sliding. (e–h) The shape of the contact interface during the sliding experiment. The solid line represents the mean shape, and the shaded area represents the standard deviation. (i–l) Contact angle as a function of azimuthal angle φ. The solid line represents the mean CA and the shaded area represents the standard deviation. The values of θ_a_ and θ_r_ are shown underneath each plot as the mean value of each respective zone, where the uncertainty is the pooled standard deviation. (m–p) Mean contact angle obtained from 10 consecutive measurements performed on the sample surface location. Each sample showcases some level of wetting adaptation, where the sample becomes less hydrophobic with each consecutive measurement.

We performed sliding measurements on four types of samples. We slide the droplet across a 1 mm range and calculate the contact angles along the CL on 20 top-view frames evenly spaced during sliding. Then, the CL shapes of the 20 frames and respective contact angles are combined to calculate the mean and standard deviation of the CL shape (Figure 3e–h) and θ_a_ and θ_r_ values along the CL (Figure 3i–l).

The samples consist of three types of nanograss, labeled #A, #B, and #C, and a self-assembled monolayer (SAM) surface (Figure 3a–d). The three types of nanograss consist of microsized silicon spikes with varying heights (5.0, 1.8, and 1.1 μm, respectively) and mean spike tip spacing (1.0, 0.27, and 0.24 μm, respectively) coated with hydrophobic fluoropolymer (see Materials and Methods section for sample information and SI Section 4 for SEM and AFM images). The SAM sample consists of a flat silicon substrate coated with octyltrichlorosilane (OTS), which maintains the same surface roughness as the silicon substrate. The SAM sample was prepared following a process described in earlier work^25^ (see also Materials and Methods section).

The mean CL is shown in Figure 3e–h in a solid line and the shaded area represents the standard deviation. The small standard deviation for each measurement shows that the surfaces are very homogeneous along the measured region. To quantify the shape of the CL we calculate its aspect ratio β = *L*/*W* for each sample, where *L* is the maximum length of the wetting interface measured along the sliding axis (*X*-axis), and *W* is the maximum width of the interface (*Y*-axis). The aspect ratios are 1.013, 1.068, 1.097, and 1.011, for samples #A, #B, #C and SAM respectively. Nanograss #A and SAM samples show an aspect ratio very close to 1, with an almost circular CL. In contrast, nanograsses #B and #D show increasingly high aspect ratios. The CLs are split into three zones, advancing (blue), transition (black), and receding (red), which are found based on the histogram of contact angle values observed around the CL (see SI Section 5).

The contact angle values obtained from the FEM calculation are shown in Figure 3i–l as a function of the azimuthal angle φ, where the solid line and shaded areas represent the mean and standard deviation, respectively. During the sliding experiment, the front of the CL (φ ∼ 180°) advances over the surface at a fixed θ_a_ (blue). Simultaneously, the CL recedes from previously wet regions at the rear of the CL (φ ∼ 0°) at the respective θ_r_ (red). The contact angle values are split into the same zones as the CL, i.e., advancing (blue), transition (black), and receding (red). The mean values observed in advancing and receding zones are shown underneath the plots in Figure 3i–l. The uncertainty represents the pooled standard deviation combining the contact angle values observed along the CL in respective zones for all 20 frames analyzed. The uncertainty can be interpreted as the standard deviation of θ_a_ and θ_r_ of the sample surface across the scanned area. The contact angles show good agreement with those obtained on our previous work^21^ in similar surfaces, which were also validated with a Digital Holography Microscope (DHM). The contact angles from our previous work are shown in Supporting Table S1, as well as the values obtained with a commercial contact angle goniometer.

During sliding, the wetting interface on the nanograss samples remains in the Cassie–Baxter state,^4^ where only the tips of the Si spikes are wet, trapping a thin air layer underneath the droplet. All three nanograss surfaces present very similar θ_a_ values near 180°. This is attributed to a similar mechanism to that observed by Schellenberger et al. on micropillared surfaces^26^ where they demonstrate that the liquid–air surface gradually bends down over each surface feature as the CL advances, resulting in a macroscopic θ_a_ near 180°. On the other hand, the CL recedes by a process of successive depinning, similarly observed also for micropillars in the same study. In this case, the receding contact angles in each nanograss sample show a decreasing trend. This can be attributed to the decreasing spike spacing in nanograsses #A through #C, i.e., increasing spike density. As a result, nanograss #A has less liquid–solid contacting area compared to #B and #C making it more hydrophobic. In the SAM surface, the wetting interface remains in the Wenzel state^27^ throughout the measurement, where no air is trapped under the droplet. The SAM sample shows much lower θ_a_ and θ_r_ values but is still in the hydrophobic regime (θ > 90°), where the contact angle hysteresis (Δθ = θ_a_ – θ_r_ = 4°) is small but is still greater than the uncertainty of each measurement.

Combining the information from the CL shape and contact angle values, we observe that all samples present θ_a_ on the whole front edge, in the range 90° ≤ φ ≤ – 90°, as shown by the blue zones in Figure 3e–h. On the other hand, the extent of the CL in the receding zone varies from sample to sample. In the nanograss surfaces, θ_r_ is approximately present in the range −60° ≤ φ ≤ 60° while in the SAM sample, the receding zone spans a bigger range, approximately −68° ≤ φ ≤ 79°. In the transition zones, the contact angle values vary continuously between θ_a_ and θ_r_.

To test the repeatability of the measurements, we performed the sliding experiment 10 times on the same sample location for each sample. The resulting mean contact angles of each measurement are shown in Figure 3m–p (mean of 20 frames per measurement). With each consecutive measurement, the samples show a progressive change in wetting properties, apart from nanograss #A which has seemingly repeatable properties. Nanograss #B and #C both show a noticeable decrease in θ_r_ with each measurement, while θ_a_ is mostly constant. On the other hand, the SAM sample shows a progressive decrease in both θ_a_ and θ_r_ with each measurement. We attribute these changes in wetting properties to the adaptation of the surface to the contact with the water droplet,^10^ where prolonged contact between the droplet and the surface leads to a decrease in the observed contact angle.

### Surface Wetting Maps

**Figure 4:**
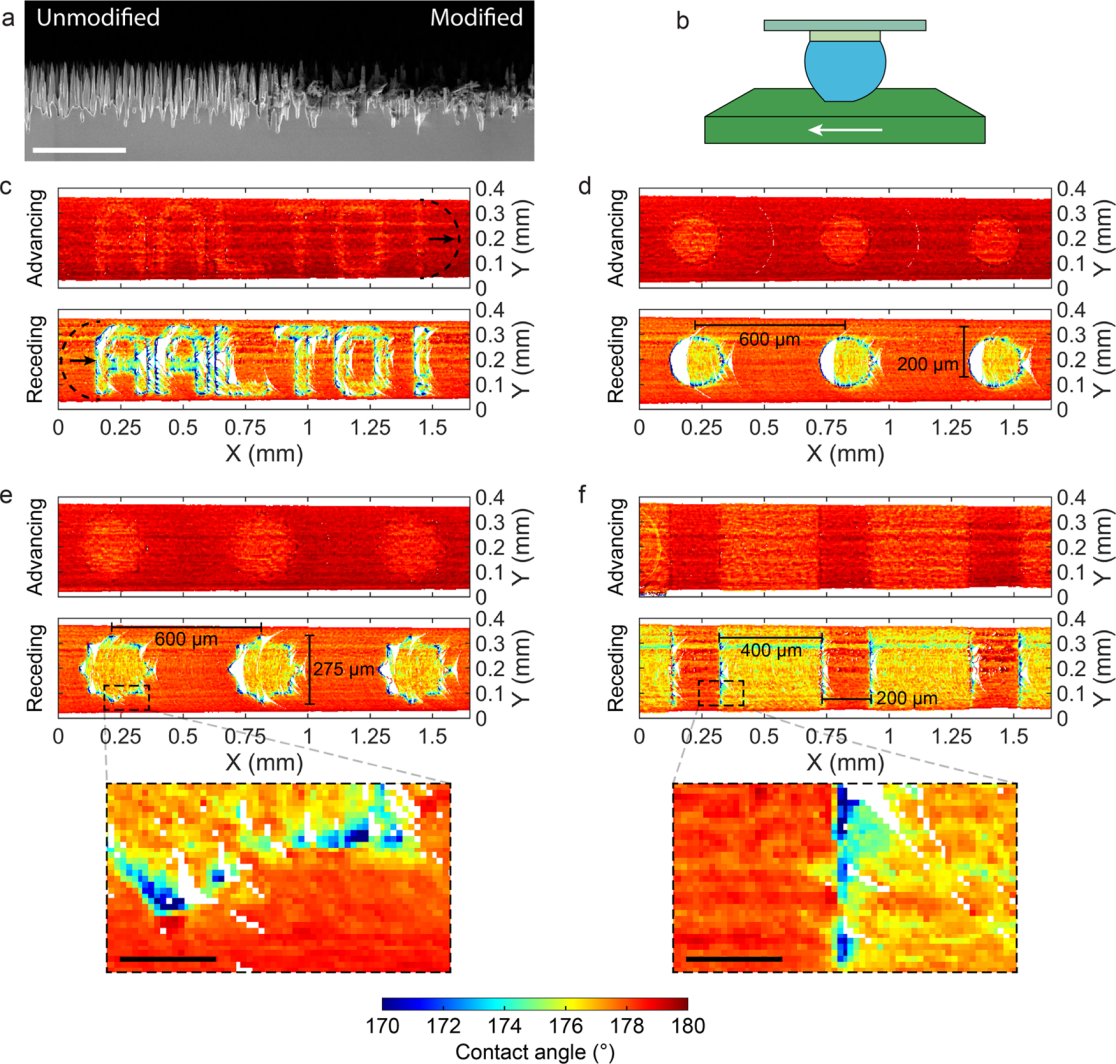
Maps of advancing and receding contact angles obtained from sliding experiments on different patterned surfaces, where the modified areas lead to different wetting properties (XY resolution: 3 μm). (a) SEM of nanograss sample, where the nonmodified nanograss area shows intact spikes compared to the modified area. (Scale bar: 5 μm) (b) Illustration of the direction of scanning for the maps shown in panels (c–f), i.e., the sample is moved right to left. (c) Map of θ_a_ (Top) and θ_r_ (Bottom) over a pattern where the modified area spells “Aalto!”. Arrows indicate the direction of the movement of the CL. (d) Wetting map on circles. (e) Wetting map on 8-tipped stars. (Inset scale bar: 50 μm) (f) Wetting map on stripes. (Inset scale bar: 50 μm) (See SI Section 6 for a more detailed view of the advancing maps).

To expand on the FEM-based method for measuring mean contact angles, we also produced point-by-point high-resolution maps of θ_a_ and θ_r_ on several patterned surfaces (Figure 4). During the sliding measurement, each point of the surface under the scanned area is visited twice by the CL. First, when the point enters the wetting interface, i.e., when the CL advances over the point and it becomes wet, θ_a_ is measured. Second, when the point leaves the interface, θ_r_ is measured. By mapping the contact angles observed to the coordinate location of the CL point on the surface, we can create maps of θ_a_ and θ_r_, shown in Figure 4.

We tested our method on four wetting patterned surfaces. The patterns are created on nanograss #A by etching the selected areas (Figure 4a) which lowers the contact angle on these zones. For all maps, the direction of the scan was made by moving the sample from the right to the left, as indicated in Figure 4b. On all patterns, the advancing CL moves smoothly across the surface, with a small wetting contrast between the modified and nonmodified zones. On the other hand, the receding CL shows greater contrast, where the modified zones show lower contact angles and the CL tends to move in steps. During such steps, the CL jumps between surface features, which may be a few micrometers apart (see Figure 4a). Moreover, we observe that the CL tends to become pinned at the edges between modified and unmodified zones and subsequently jump bigger gaps to a new equilibrium position (see Video S1). This results in regions where the contact angle information is missing, shown as empty spots in the receding maps. The maps also reveal horizontal line patterns, such as that seen in the receding map of Figure 4f. We attribute these to surface debris that are pinned to the CL.^6^ These accumulate at the air–water interface near the CL and influence the perceived wetting properties.

The first wetting pattern in Figure 4c shows the text “Aalto!”. While the advancing CL moves smoothly over the wetting features, the receding CL shows that the interaction between the droplet and the pattern has many pinning locations on the edges between modified and nonmodified zones. The second pattern, Figure 4d, shows the wetting maps over circularly shaped zones. The receding CL is strongly pinned at the left edge of the circles. When the stresses in the surface of the droplet are large enough, the receding CL depins and jumps over a gap of approximately 60 μm, leaving a significant section of the circle without receding contact angle information. The depinning process happens in less than 10 ms, given that the top-view videos are acquired at 100 fps. Figure 4e shows wetting maps over 8-tip star-shaped zones, where the tips of the stars form a 90° angle. In this sample, the pinning of the receding CL mostly occurs on the tips of the stars. The three stars in the scanned area show a noticeable difference in the wetting behavior. In the left star, the depinning of the receding CL is less abrupt, resulting in smaller gaps in the map, while the other two, middle and right-most, show more chaotic motion of the CL. Figure 4f shows the wetting maps over an area featuring modified zones in the shape of vertical stripes. It is observed that the CL is pinned at the edge between zones, with some depinning events observed.

The spatial resolution of the method is limited by the ability to resolve changes in the position of the CL. In our experimental setup, the resolution of the top-view camera is ∼0.7 μm. However, the spacing between spikes in the nanograss is between 1 and 3 μm, and the CL moves in steps of similar size. The sample is moved at 100 μm/s, which results in a displacement of 1 μm between each frame. For the experiments in Figure 4, the wetting maps were plotted with a resolution of 3 μm, i.e., the value of each pixel represents the average of contact angles observed within that 3 × 3 μm^2^. A smaller resolution can be chosen if the surface has smaller features.

The wetting maps showcase the great sensitivity of the technique. The modified and nonmodified zones show very small differences in wetting properties, mostly between 175 and 180° with small contact angle hysteresis (see Figure 4 color-bar). Nonetheless, the method is able to reliably differentiate between zones, even with the advancing contact angle. Moreover, the wetting behavior showcases rich phenomena such as pinning of the receding CL on the edges between zones, which greatly influences the motion of the droplet.

## Conclusions

The method presented estimates advancing and receding contact angle values along the contact line of a droplet probe sliding on hydrophobic surfaces with unprecedented resolution. The approach combines through-droplet imaging with FEM modeling of the shape of the droplet to obtain detailed information about the wetting properties of hydrophobic surfaces. The technique showcases how a millimeter-sized droplet can be used to obtain wetting information on the micrometer scale, the same scale as the surface features. The ability to measure the contact angles along the CL of sliding droplets provides direct point-by-point maps of the wetting properties of inhomogeneous surfaces, where previous techniques provided only average values. This ability is critical in both the development of novel wetting surface treatments and the study of natural surfaces.

The method was used to characterize three types of nanograss superhydrophobic surfaces and a hydrophobic SAM sample. The measurements revealed the great homogeneity of the surfaces. The mean CL shape and the mean contact angle values along the CL were calculated, showing great homogeneity of these surfaces. The data show that during the sliding experiments, the advancing contact angle profile along the CL is observed throughout most of the front of the CL while the receding contact angle is observed on a large extent of the back of the CL, with transition zones in between. Moreover, the method reveals important adaptation phenomena that may directly affect wetting measurement methods in general, where the longer the liquid interacts with the test surface, the lower the contact angle tends to become.

To complement the results, the ability to map the contact angle values along the contact line was used to produce detailed wetting maps of the patterned surfaces with a resolution of 3 μm. On these maps, a pair of advancing and receding contact angle values are obtained at each point of the sample. The maps are able to distinguish contact angles between zones with very similar wetting properties, despite the values being very similar between zones. The boundary between the modified and nonmodified areas forms locations where pinning of the CL is likely to occur. In turn, such pinning may lead to abrupt CL movements during sliding.

The insights provided by this technique have the potential to contribute to the growing body of knowledge on surface wetting characterization and may inform future experimental design and interpretation of the development of highly sensitive hydrophobic and superhydrophobic surfaces.

## Materials and Methods

### Measurement Procedure

Prior to each measurement, a water droplet is formed on the holding disk with a volume above the target of 0.5 μL. The volume is estimated using a custom image analysis algorithm at the start of the measurement. The sample is then moved such that the droplet is above the measurement site. The measurement is carried out in three successive motions. First, the sample stage moves upward, compressing the droplet by a set amount (100 μm on nanograss samples and patterned surfaces and 5 μm on SAM). Second, the sample stage moves laterally for a set distance (1 mm for the measurements of Figures 3 and 4 mm for the measurements of Figure 4) at a speed of 100 μm/s. Third, the sample stage is moved downward, decompressing the droplet, until the droplet detaches from the surface. All measurements were performed at a temperature of 24–25 °C, with a relative humidity of 15% in the measurements of Figure 3 and 69% in the measurements of Figure 4. The measurements in Figure 3m–p were made consecutively. The droplet was refilled and controlled to 0.5 μL between measurements to account for the loss in volume due to evaporation.

### Apparatus

The top-view and side-view images were obtained by cameras operating at 100 frames-per-second and at a resolution of 1464 × 1464 px^2^, model BFS-U3–28S5M C (Flir LLC) using a variable zoom lens (VZM 600i, Edmund Optics Inc.) with 1–6× magnification. To illuminate the wetting interface, a 15 mm 50R/50T standard cube beam splitter (Edmund Optics Inc.) was assembled on the top-view lens. The light source used was model OSL2, with a collimating lens, model OSL2COL (Thorlabs Inc.). A three-axis precision motorized stage is used to move the sample, models M-404.8PD, M-122.2DD, and M-111.1DG, for the *X*, *Y*, and *Z* axes, respectively (Physik Instrumente GmbH, Germany). The top-view camera was mounted on a precision motorized stage, model M-122.2DD (Physik Instrumente GmbH, Germany), for focus tracking. The relative *Z*-axis sample displacement was measured using an interferometer laser, model IDS3010, with a fiberoptic sensor head, model D4/F17 (Attocube Systems AG, Germany). A reflective silicon wafer cutout was placed on the sample stage, providing a reflective surface for the interferometer. The liquid droplet probe was formed with a nanoliter dispenser PipeJet from BioFluidix GmbH, Germany. Type 1 ultrapure water was used in all measurements, with a resistivity of 18.2 MΩ·cm, obtained with Direct-Q 3 UV Water Purification System, Milli-Q. Several custom 3D-printed parts were produced using a stereolithography printer (model SL1, Prusa Research a.s., Czech Republic) from liquid resin Strong-X, by Liqcreate, Netherlands. To collect the data, a data acquisition board was used, model NI USB 6363 (National Instruments Inc.). The setup was controlled with custom software.

### Image Analysis for Contact Line Identification

The top-view image analysis is done in MATLAB to identify the CL in two steps. In the first step, a flat-field correction is applied to reduce the effect of shadows cast by defects in the droplet-holding disk. A reference image was obtained on a flat surface FF and then combined with the raw top-view frame *R*. The corrected image *C* is produced by the following relation
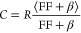
2where β is a constant bias which accounts for the difference in the brightness of the light source during acquisition of the flat-field and the measurements (see SI Section 2 for more details). ⟨FF + β⟩ is the mean pixel value of the bias-corrected flat-field image.

In the second step, CL is identified. The corrected grayscale image is converted into a binary image by applying a predetermined threshold value. In this binary image, regions of connected pixels are identified. The background is identified as the largest such region, which is then removed from the binary image. The wetting interface is the largest remaining region which is then selected, and any discontinuities within it are filled to create a continuous shape. The CL is taken as the perimeter pixels of the shape representing the wetting interface. More detailed explanation and example code can be found in GitHub.^28^

### Surface Evolver Calculations

To find the shape of the droplet for any given frame of the measurement, the shape of the droplet is initialized to a simplified mesh connecting the CL and the droplet-holding disk, which is automatically defined by a MATLAB script. The shape of the CL is extracted from the top-view images, downsampled to 500 points, and placed in the *XY* plane at *Z* = 0, in a fixed position. The droplet-holding disk is created as 500 points in a circle with a 511 μm radius (radius of the droplet-holding disk) at *Z* = *h* also in a fixed position, where *h* is the height of the droplet, i.e., sample-to-disk distance. The points that define the droplet-holding disk and the CL are directly connected to form the initial state of the water–air interface. For each frame, the MATLAB code generates a Surface Evolver Simulation. Then, Surface Evolver iteratively refines and moves the points defining the water–air interface to minimize the total energy of the system until the final shape of the droplet is obtained (Figure 2b,c). More detailed explanation and source code can be found in GitHub.^28^

### Silicon Nanograss Coated with Fluoropolymer (#A, #B, and #C)

The silicon nanograss was produced by a maskless cryogenic deep reactive ion etching process using an Oxford Plasmalab System 100 on a 4-in silicon wafer (⟨100⟩, p-type boron doped, >1 Ω·cm). The process parameters were 1000 W of ICP forward power, a temperature of −110 °C, 10 mTorr of pressure, and 7 min of etching time. Varying etching gas flow rates and forward powers were utilized to obtain the different nanograss morphologies. For nanograss #A, #B, and #C, the SF_6_ gas flow rate was 40.0, 35.3, and 32.9 sccm, respectively; the O_2_ gas flow rate was 18.0, 22.8, and 25.1 sccm, respectively; and the forward power was 6, 5, and 4 W, respectively. All etched silicon nanograss samples were coated with a thin fluoropolymer film for superhydrophobicity by plasma-enhanced chemical vapor deposition (PECVD) using an Oxford Plasmalab 80+ with 100 sccm of CHF_3_ for 5 min under 250 mTorr of pressure and 50 W of forward power.

### Self-Assembled Monolayer

To produce the SAM sample a silicon wafer (⟨100⟩, undoped, >10,000 Ω·cm) substrate was first cleaned and activated with a 30 min UV–O_3_ treatment (BioForce Nanosciences), after which it was immediately transferred into a preheated (60 °C) atomic layer deposition reactor (Savannah S200, Veeco) for the SAM growth. The reactor was first pumped down to vacuum (base pressure ca. 7 Pa) and allowed to stabilize for 30 min under 20 sccm N_2_ carrier gas flow. Then a 15 ms water pulse was applied followed by 10 s purge time. Next, the carrier gas flow was turned off, the reactor disconnected from the vacuum line, and octyltrichlorosilane (OTS, 97%, Sigma-Aldrich) dosed (50 ± 10 Pa) into the reactor. Periodically, the dose was purged off from the reactor to remove the generated byproduct HCl, which was followed by a fresh OTS dose (details in ref (25)). The total time of the deposition of the OTS was 24 h.

### Nanograss Patterned Surfaces

The wettability patterned nanograss surfaces were fabricated from the starting point of nanograss #A. First, a PECVD silicon dioxide film was deposited using Oxford Plasmalab 80+ in 300 °C temperature, 1000 mTorr pressure, 20 W power, and the gas flows were 8.5 sccm SiH_4_, 710 sccm N_2_O and 161.5 sccm N_2_ for 8 min (≈500 nm). The oxide layer was then patterned by UV lithography using AZ4562 resist. The photoresist pattern acted as a mask during RIE etching of the oxide Oxford Plasmalab80+ 200 mTorr pressure, 30 W power, 25 sccm CHF_3_, 25 sccm Ar for 18 min. The photoresist was stripped in acetone. The oxide mask was then used to selectively protect the silicon nanograss surface from modification by a silicon etch, Oxford Plasmalab 80+ 30 mTorr pressure, 100 W forward power, 100 sccm SiSF_6_, and 1 min etching time. The oxide mask was then removed in a buffered HF.

## Supporting Information Available

Experimental setup; flat-field correction; volume and sample-to-disk height estimation; SEM and AFM measurements; estimating contact angle from histogram; advancing wetting maps at smaller *z*-axis scale. It also includes supporting Table S1–contact angles on various surfaces measured with different methods (PDF)
Scanning of the four patterned surfaces discussed in Figure 4, with patterns for “Aalto!”, Stars, Circles, and Stripes. The videos include an overlay of the CL detected with our MATLAB code (MP4)

## References

[ref1] Barthlott W.; Neinhuis C. Purity of the Sacred Lotus, or Escape from Contamination in Biological Surfaces. Planta 1997, 202 (1), 1–8. 10.1007/s004250050096.

[ref2] Bhushan B.; Nosonovsky M. The Rose Petal Effect and the Modes of Superhydrophobicity. Philos. Trans. R. Soc., A 2010, 368 (1929), 4713–4728. 10.1098/rsta.2010.0203. 20855317

[ref3] Wagner T.; Neinhuis C.; Barthlott W. Wettability and Contaminability of Insect Wings as a Function of Their Surface Sculptures. Acta Zool. 1996, 77 (3), 213–225. 10.1111/j.1463-6395.1996.tb01265.x.

[ref4] Cassie A. B. D.; Baxter S. Wettability of Porous Surfaces. Trans. Faraday Soc. 1944, 40, 546 10.1039/tf9444000546.

[ref5] Park K.-C.; Chhatre S. S.; Srinivasan S.; Cohen R. E.; McKinley G. H. Optimal Design of Permeable Fiber Network Structures for Fog Harvesting. Langmuir 2013, 29 (43), 13269–13277. 10.1021/la402409f. 23895249

[ref6] Geyer F.; D’Acunzi M.; Sharifi-Aghili A.; Saal A.; Gao N.; Kaltbeitzel A.; Sloot T.-F.; Berger R.; Butt H.-J.; Vollmer D. When and How Self-Cleaning of Superhydrophobic Surfaces Works. Sci. Adv. 2020, 6 (3), eaaw9727 10.1126/sciadv.aaw9727. 32010764 PMC6968945

[ref7] Mastrangeli M.; Zhou Q.; Sariola V.; Lambert P. Surface Tension-Driven Self-Alignment. Soft Matter 2017, 13 (2), 304–327. 10.1039/C6SM02078J. 27905611

[ref8] Sariola V.; Jääskeläinen M.; Zhou Q. Hybrid Microassembly Combining Robotics and Water Droplet Self-Alignment. IEEE Trans. Rob. 2010, 26 (6), 965–977. 10.1109/TRO.2010.2066830.

[ref9] Huhtamäki T.; Tian X.; Korhonen J. T.; Ras R. H. A. Surface-Wetting Characterization Using Contact-Angle Measurements. Nat. Protoc. 2018, 13 (7), 1521–1538. 10.1038/s41596-018-0003-z. 29988109

[ref10] Butt H.-J.; Liu J.; Koynov K.; Straub B.; Hinduja C.; Roismann I.; Berger R.; Li X.; Vollmer D.; Steffen W.; Kappl M. Contact Angle Hysteresis. Curr. Opin. Colloid Interface Sci. 2022, 59, 101574 10.1016/j.cocis.2022.101574.

[ref11] Sun Y.; Jiang Y.; Choi C.-H.; Xie G.; Liu Q.; Drelich J. W. The Most Stable State of a Droplet on Anisotropic Patterns: Support for a Missing Link. Surf. Innovations 2018, 6 (3), 133–140. 10.1680/jsuin.17.00064.

[ref12] Govindha A. H.; Katre P.; Balusamy S.; Banerjee S.; Sahu K. C. Counter-Intuitive Evaporation in Nanofluids Droplets Due to Stick-Slip Nature. Langmuir 2022, 38 (49), 15361–15371. 10.1021/acs.langmuir.2c02590. 36459485

[ref13] Extrand C. W.; Kumagai Y. Liquid Drops on an Inclined Plane: The Relation between Contact Angles, Drop Shape, and Retentive Force. J. Colloid Interface Sci. 1995, 170 (2), 515–521. 10.1006/jcis.1995.1130.

[ref14] Sáenz P. J.; Wray A. W.; Che Z.; Matar O. K.; Valluri P.; Kim J.; Sefiane K. Dynamics and Universal Scaling Law in Geometrically-Controlled Sessile Drop Evaporation. Nat. Commun. 2017, 8 (1), 14783 10.1038/ncomms14783. 28294114 PMC5355953

[ref15] Vuckovac M.; Latikka M.; Liu K.; Huhtamäki T.; Ras R. H. A. Uncertainties in Contact Angle Goniometry. Soft Matter 2019, 15 (35), 7089–7096. 10.1039/C9SM01221D. 31453607

[ref16] Liu K.; Vuckovac M.; Latikka M.; Huhtamäki T.; Ras R. H. A. Improving Surface-Wetting Characterization. Science 2019, 363, 1147–1148. 10.1126/science.aav5388. 30872505

[ref17] Wang D.; Jiang Y.; Zhu Z.; Yin W.; Asawa K.; Choi C. H.; Drelich J. W. Contact Line and Adhesion Force of Droplets on Concentric Ring-Textured Hydrophobic Surfaces. Langmuir 2020, 36, 2622–2628. 10.1021/acs.langmuir.9b03953. 32133857

[ref18] Saal A.; Straub B. B.; Butt H.-J.; Berger R. Pinning Forces of Sliding Drops at Defects. Europhys. Lett. 2022, 139 (4), 47001 10.1209/0295-5075/ac7acf.

[ref19] Papadopoulos P.; Deng X.; Mammen L.; Drotlef D. M.; Battagliarin G.; Li C.; Müllen K.; Landfester K.; Del Campo A.; Butt H. J.; Vollmer D. Wetting on the Microscale: Shape of a Liquid Drop on a Microstructured Surface at Different Length Scales. Langmuir 2012, 28 (22), 8392–8398. 10.1021/la300379u. 22578130

[ref20] Kashaninejad N.; Nguyen N.-T.; Chan W. K. The Three-Phase Contact Line Shape and Eccentricity Effect of Anisotropic Wetting on Hydrophobic Surfaces. Soft Matter 2013, 9 (2), 527–535. 10.1039/C2SM26963E.

[ref21] Vieira A.; Cui W.; Jokinen V.; Ras R. H. A.; Zhou Q. Through-Drop Imaging of Moving Contact Lines and Contact Areas on Opaque Water-Repellent Surfaces. Soft Matter 2023, 19, 2350–2359. 10.1039/D2SM01622B. 36880312 PMC10053025

[ref22] Vieira A.; Zhou Q. Multimodal Sensing Transparent Droplet Probe for Characterization of Superhydrophobic Surfaces. IEEE Sens J. 2023, 23 (15), 17462–17469. 10.1109/JSEN.2023.3288333.

[ref23] Seibert J. A.; Boone J. M.; Lindfors K. K. In Flat-Field Correction Technique for Digital Detectors, SPIE Proceedings; SPIE, 1998; p 348.

[ref24] Brakke K. A. The Surface Evolver. Exp. Math. 1992, 1 (2), 141–165. 10.1080/10586458.1992.10504253.

[ref25] Lepikko S.; Jaques Y. M.; Junaid M.; Backholm M.; Lahtinen J.; Julin J.; Jokinen V.; Sajavaara T.; Sammalkorpi M.; Foster A. S.; Ras R. H. A. Droplet Slipperiness despite Surface Heterogeneity at Molecular Scale. Nat. Chem. 2023, 1–8. 10.1038/s41557-023-01346-3. 37872419 PMC10997520

[ref26] Schellenberger F.; Encinas N.; Vollmer D.; Butt H. J. How Water Advances on Superhydrophobic Surfaces. Phys. Rev. Lett. 2016, 116 (9), 096101 10.1103/PhysRevLett.116.096101. 26991185

[ref27] Wenzel R. N. Resistance of Solid Surfaces to Wetting by Water. Ind. Eng. Chem. 1936, 28 (8), 988–994. 10.1021/ie50320a024.

[ref28] Vieira A. Codes for Measuring Contact Angles along Contact Line, https://github.com/TheAVieira/disk-to-flat-sample_SurfaceEvolverStudy.

