# Supplementary material for: Through-Drop Imaging of Liquid–Solid Interfaces: From Contact Angle Variations Along the Droplet Perimeter to Mapping of Contact Angles Across a Surface
Source: Langmuir. 2024 Apr 15;40(17):9059–67. doi: 10.1021/acs.langmuir.4c00414 (PMC11072716; doi:10.1021/acs.langmuir.4c00414)
Supplement: Supplementary file 1 — la4c00414_si_001.pdf [file la4c00414_si_001.pdf]

Supporting Information

Through-drop imaging of liquid-solid interfaces: From contact angle variations along the droplet perimeter to mapping of contact angles across a surface

Arthur Vieira, Ville Jokinen, Sakari Lepikko, Robin H. A. Ras and Quan Zhou**

Corresponding authors

Arthur Vieira – Department of Electrical Engineering and Automation, School of Electrical Engineering, Aalto University, Maarintie 8, 02150 Espoo, Finland.

E-mail: arthur.vieira@aalto.fi

Quan Zhou – Department of Electrical Engineering and Automation, School of Electrical Engineering, Aalto University, Maarintie 8, 02150 Espoo, Finland.

E-mail: quan.zhou@aalto.fi

Authors

Ville Jokinen – Department of Chemistry and Materials Science, School of Chemical Engineering, Aalto University, Tietotie 3, 02150 Espoo, Finland

Sakari Lepikko – Department of Applied Physics, Aalto University, Espoo, Finland.

Centre of Excellence in Life-Inspired Hybrid Materials (LIBER), Aalto University, 02150 Espoo, Finland

Robin H. A. Ras – Department of Applied Physics, Aalto University, Espoo, Finland.

Centre of Excellence in Life-Inspired Hybrid Materials (LIBER), Aalto University, 02150 Espoo, Finland

Table of Contents

Section 1 – Experimental setup 3

Section 2 – Flat-field correction 4

Section 3 - Volume and sample-to-disk height estimation 5

Section 4 – SEM and AFM measurements 6

Section 5 – Estimating contact angle from histogram 7

Section 6 – Advancing wetting maps at smaller z-axis scale 8

Supporting Tables 9

Section 1 – Experimental setup

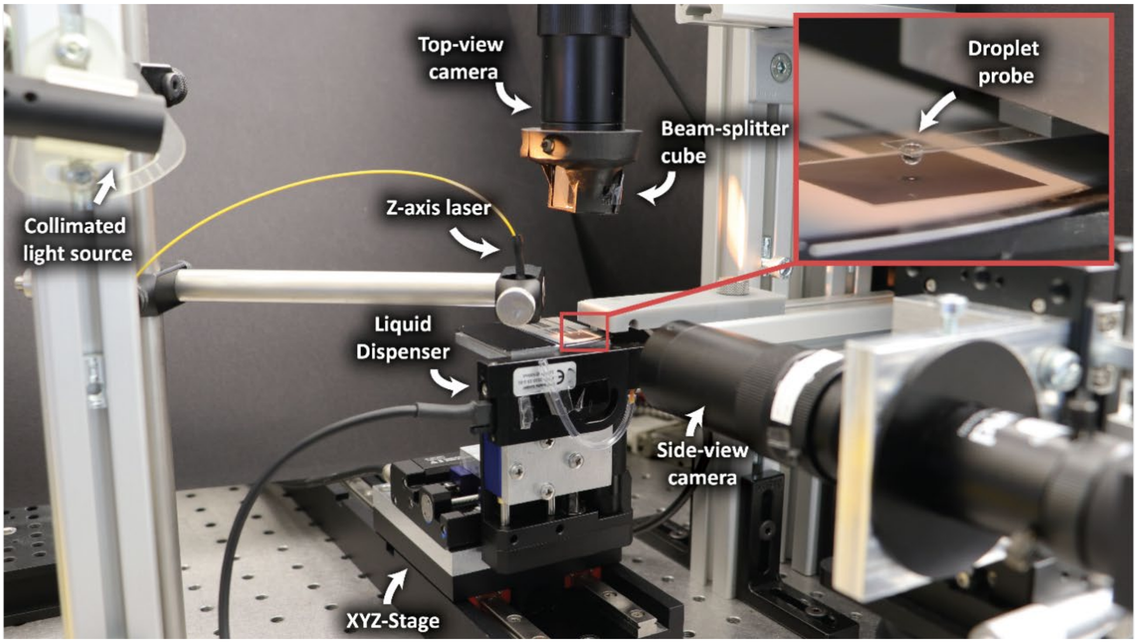

Fig. S1 Photograph of the experimental setup. The inset shows the droplet probe consisting of a water droplet hanging from the transparent holding disk.

Section 2 – Flat-field correction

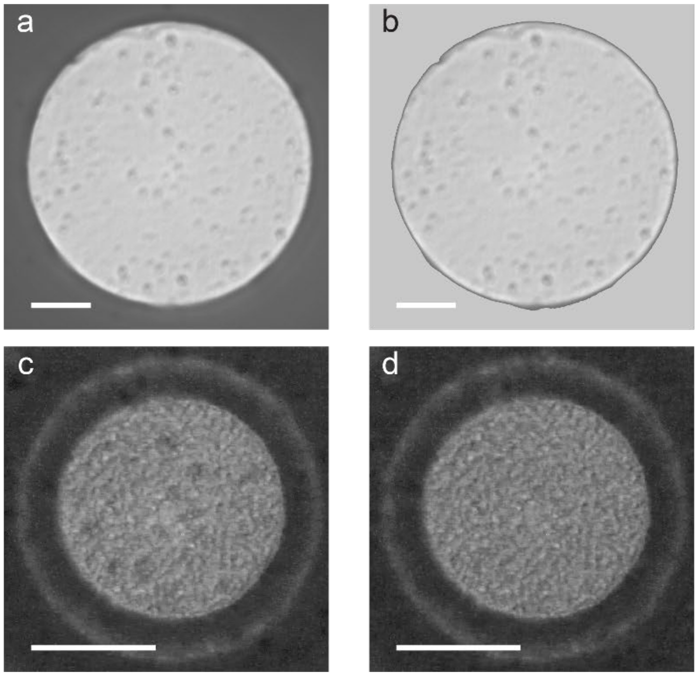

Fig. S2 Flat-field correction process. **(a)** Flat-field image obtained on a bare silicon wafer. The field-of-view includes the whole droplet-holding-disk (Disk diameter ~ 1 mm) **(b)** Flat-field image after isolating the background such that the zone outside the droplet holding disk will not affect the processed images. **(c)** Example of raw top-view frame where darker spots are caused by shadows cast from the imperfections of the droplet-holding-disk. (Wetting interface diameter ~ 365 μm) **(d)** Corrected image. Scale bars: 200 μm

The top-view images of the wetting interface appear with shadowed zones due imperfections in the SU-8 droplet-holding-disk resulting from manufacturing. These shadows may interfere with the machine vision identification of the wetting interface, especially if the shadows are cast near the edge of the wetting interface. For this reason, a flat-field correction is applied to top-view frames, where a reference image is used to remove the imperfections from the image. The reference image is acquired on a flat piece of silicon with care to ensure that the disk-sample height and top-camera focus are the same as during experiments (Fig. S2a). Under such conditions, the variations in image brightness are due only to shadows cast by the imperfections in the disk. Before correction, the region of reference image outside the drop-holding-disk, i.e. dark background in Fig. S2a, is replaced with the mean of the interior region, which creates a normalized reference image as shown in Fig. S2b. When applied, the normalized reference image will only affect the region inside the disk. We note that the reference image provides a bigger area for correction than the typical size of the wetting interface during the experiment (see scale bars in Fig. 2S). Fig. S2c shows an example image of the wetting interface in nanograss before correction, and the resulting image after correction, Fig. S2d.

Section 3 - Volume and sample-to-disk height estimation

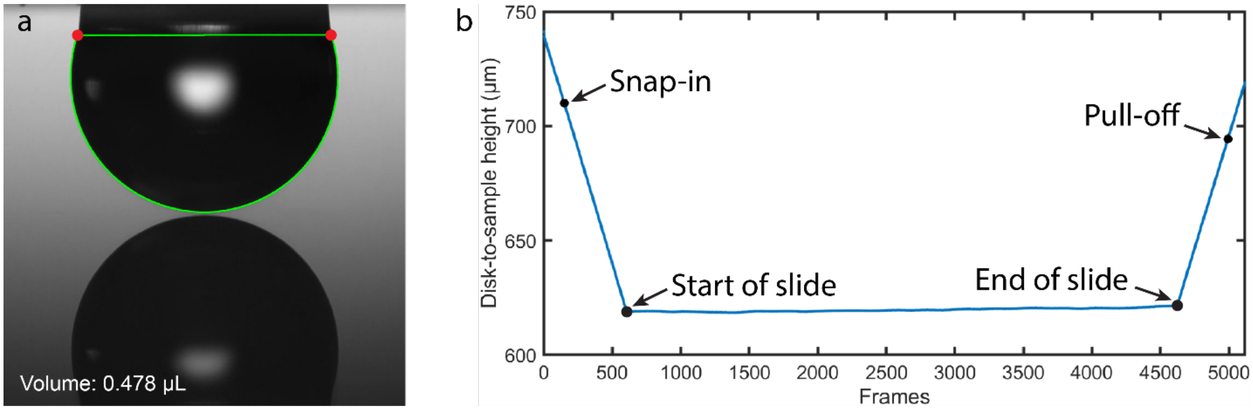

Fig. S3 Droplet volume and disk-to-sample height estimation. **(a)** Volume is identified by machine vision from the outline of the droplet (green). The disk edge points, marked in red, are used by the machine vision algorithm to exclude the disk from the droplet. **(b)** Disk-to-sample height profile during sliding measurement. The snap-in marks the moment when droplet first touches the sample, and pull-off marks the moment when droplet detaches from the sample.

To perform Surface Evolver simulations, it is necessary to know the disk-to-sample height h and the volume of the droplet for each top-view frame simulated. To estimate the volume of the droplet during the measurement, we first measure the volume 100 frames before snap-in (moment when droplet first touches the sample), and 50 frames after pull-off, directly from side-view. Fig. S3a shows the result of machine vision detection of the droplet profile from which the volume is estimated. The edge points of the disk, marked red, are found through template matching, where the templates for the left and right sides of the drop are created manually. The outline of the droplet is found through binary threshold and the disk edge points are used to exclude the droplet-holding-disk from the outline of the droplet. Before contacting the sample, the droplet is assumed to have spherical shape. The volume is then calculated as the sum of the volume of individual horizontal slices (indexed with i), which have diameter D_i obtained direct from the droplet profile, and height Δh corresponding to the size of a pixel.

$$V = \sum_i \pi \left(\frac{D_i}{2}\right)^2 \Delta h. \tag{1}$$

After finding the volume for the two moments before and after the experiment, the volume during experiment is estimated by linear interpolation, which accounts for droplet evaporation.

To estimate the disk-to-sample height (Fig. S3b) a mirrored surface (silicon wafer cutout) is placed in parallel with the sample on the sample-stage. A laser interferometer measures the relative displacement of the z-axis of the sample stage. The Z-axis displacement data serves as a relative measure of the sample's vertical position. The height of the droplet, calculated at the moment of snap-in, is used to offset this data and transforms the Z-axis data into an absolute measure of the distance between the disk and the sample for the frames between snap-in and pull-off. The droplet heigh is calculated geometrically based on a spherical cap with a known volume attached to a disk of known radius (~511 µm)

Section 4 – SEM and AFM measurements

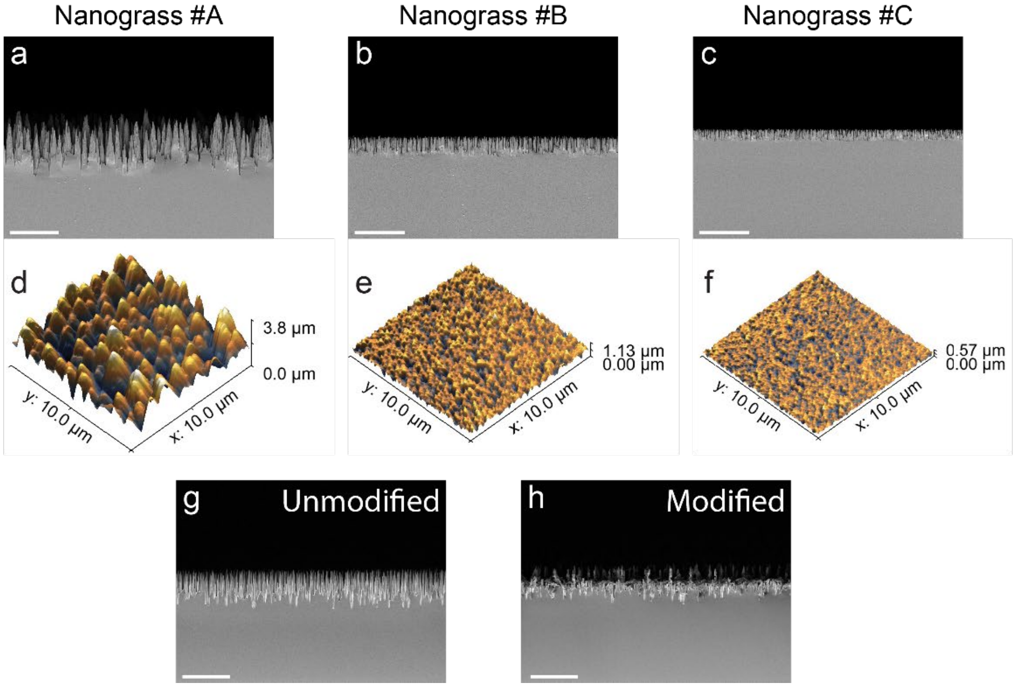

Fig. S4 SEM and AFM images of the nanograss samples. **(a-c)** SEM images of nanograsses used in the data in Fig. 3 of main text. Silicon nanograss samples were cut and coated with 5 nm of Gold-Palladium using a Leica EM ACE600 high vacuum sputter coater. Images were taken with a Zeiss Sigma VP SEM, with an acceleration voltage of 2 kV, in high vacuum mode using an in-lens secondary electron detector. Scale bar: 5 µm. **(d-f)** AFM images. AFM images of silicon nanograss samples were obtained using a Dimension Icon AFM (Bruker Corporation) in ScanAsyst mode with a ScanAsyst-Air probe. The height of the spikes was measured manually from SEM images using Adobe Illustrator. The spike spacing was determined from AFM images using Gwyddion software. **(g-h)** SEM images of nanograsses used in the data in Fig. 4 of the main text. The unmodified sample shows intact nanograss spikes. The modified sample shows partial etching of nanograss spikes. The samples were imaged without metal coating with a Zeiss Sigma VP SEM, with an acceleration voltage of 2 kV. Scale bar: 5 µm.

Section 5 – Estimating contact angle from histogram

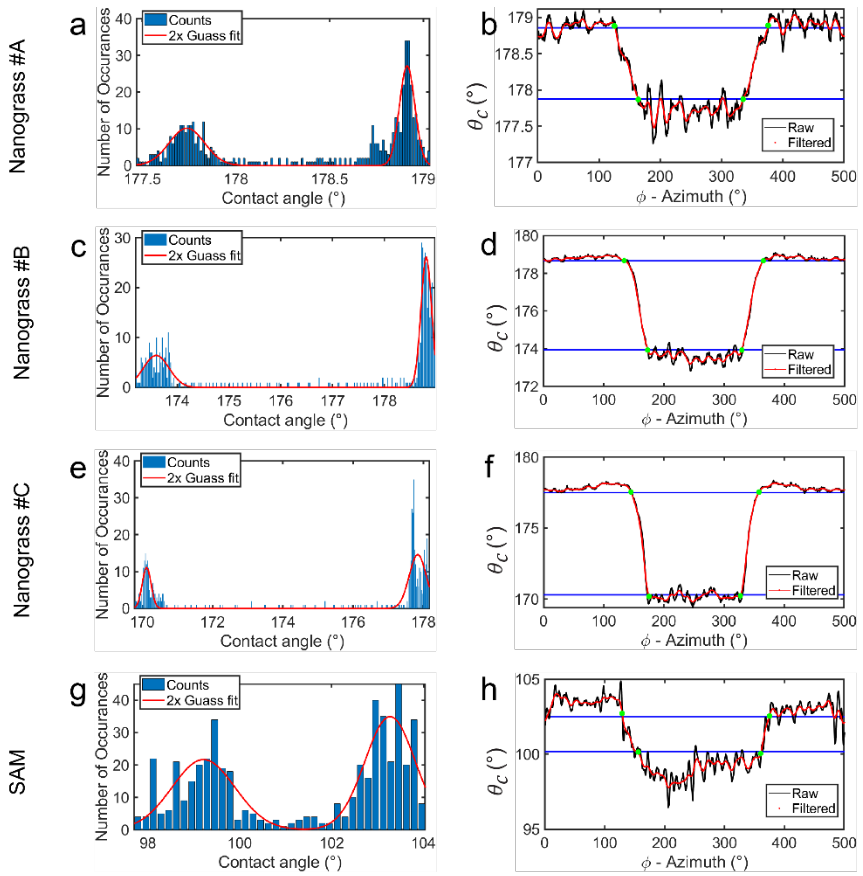

Fig. S5 A double gaussian fit allows segmenting the CL and the respective contact angles into three zones, advancing, transition and receding, as seen in Fig. 3 of the manuscript. **(a)** Histogram of the contact angle values observed around the CL (blue) on nanograss #A. A double gaussian fit (red) is performed to estimate the mean and standard deviation of advancing and receding contact angles, $\bar{\theta}_a \pm \sigma_{\theta_a}$ and $\hat{\theta}_r \pm \sigma_{\theta_r}$ respectively. **(b)** The contact angle (black) is first filtered (red). A top threshold line (blue) is set to $\bar{\theta}_a - \sigma_{\theta_a}$, while the bottom is set to $\bar{\theta}_r + \sigma_{\theta_r}$. Four azimuth coordinates are found (at the green points), which will be used to split the data into the three zones. The top-left point is found as the right-most coordinate with azimuth less than 270 °, where the filtered data intersects the upper threshold. The top-right point is found as the left-most coordinate with azimuth greater than 270 °. Similarly, the bottom two points are found as the left-most and right-most points where the filtered data intersects the lower threshold. **(c, e, g)** Contact angle histograms for nanograss #B, nanograss #C and SAM samples respectively. **(d, f, h)** Contact angle values (black and red), threshold lines (blue) and respective zone delimiting points (green) for nanograss #B, nanograss #C and SAM samples respectively.

Section 6 – Advancing wetting maps at smaller z-axis scale

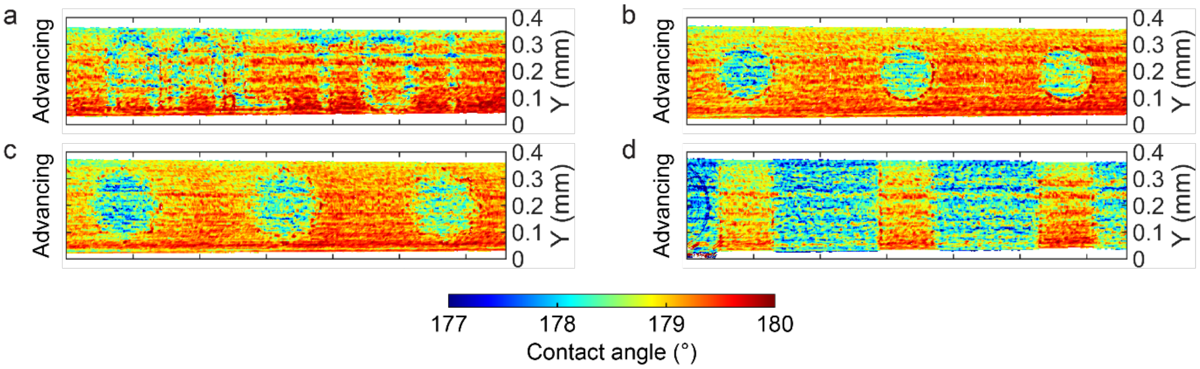

Fig. S6 Maps of advancing contact angle obtained from sliding experiments. Same as results in Fig. 4 of the manuscript. These are shown with different contact angle color range, from 177° to 180°, for better visualization of surface wetting features. **(a)** Map of θ_a over a pattern where the modified area spells "Aalto!". **(b)** Wetting map on circles. **(c)** Wetting map on 8-tipped stars. **(d)** Wetting map on stripes.

Supporting Tables

Table S1 Contact angle (mean ± standard deviation) for each type of sample measured with different methods. First column shows values obtained with the technique presented in main text. Second column show values obtained with commercial contact angle goniometer, Attension® Theta Lite optical tensiometer (Biolin Scientific). Third and forth columns show results from our previous publication on same samples: using an analytical model based on axisymmetric Young-Laplace equation; and also through direct measurement using a Digital Holographic Microscope (DHM).

	**This work**	**Theta Lite**	**Analytical model***	**DHM***
**Nanograss #A**	$\theta_a = 178.9 \pm 0.4\,°$<br>$\theta_r = 177.7 \pm 0.5\,°$	$\theta_a = 171 \pm 4\,°$<br>$\theta_r = 170 \pm 6\,°$	$\theta_a = 178.9 \pm 0.2\,°$<br>$\theta_r = 177.7 \pm 0.5\,°$	$\theta_a = 179.2 \pm 0.3\,°$<br>$\theta_r = 178.8 \pm 0.5\,°$
**Nanograss #B**	$\theta_a = 178.8 \pm 0.4\,°$<br>$\theta_r = 173.6 \pm 0.9\,°$	$\theta_a = 171 \pm 1.7\,°$<br>$\theta_r = 172 \pm 1.0\,°$	$\theta_a = 178.8 \pm 0.2\,°$<br>$\theta_r = 171.9 \pm 0.7\,°$	$\theta_a = 179.1 \pm 0.2\,°$<br>$\theta_r = NA$**
**Nanograss #C**	$\theta_a = 177.9 \pm 0.5\,°$<br>$\theta_r = 170 \pm 1.0\,°$	$\theta_a = 170 \pm 2\,°$<br>$\theta_r = 167 \pm 3\,°$	$\theta_a = 178.0 \pm 0.3\,°$<br>$\theta_r = 164.3 \pm 0.4\,°$	$\theta_a = 178.3 \pm 0.5\,°$<br>$\theta_r = NA$**
**SAM**	$\theta_a = 103.2 \pm 0.9\,°$<br>$\theta_r = 99 \pm 1.1\,°$	$\theta_a = 104.7 \pm 0.5\,°$<br>$\theta_r = 93.2 \pm 0.7\,°$	NA	NA
**Patterns unmodified zone**	$\theta_a = 179.2 \pm 0.2\,°$<br>$\theta_r = 178.0 \pm 0.3\,°$	$\theta_a = 172 \pm 3\,°$<br>$\theta_r = 170 \pm 1.4\,°$	NA	NA
**Patterns modified zone**	$\theta_a = 178.2 \pm 0.4\,°$<br>$\theta_r = 176.9 \pm 0.5\,°$	$\theta_a = 173 \pm 1.9\,°$<br>$\theta_r = 173 \pm 2\,°$	NA	NA

* Values from previous publication, where the mean and standard deviation were calculated for 10 consecutive measurements. Measurements were made on different wafers, manufactured with same recipe. - Vieira, A.; Cui, W.; Jokinen, V.; Ras, R. H. A.; Zhou, Q. Through-Drop Imaging of Moving Contact Lines and Contact Areas on Opaque Water-Repellent Surfaces. *Soft Matter* **2023**. https://doi.org/10.1039/D2SM01622B.

** Values were not possible to measure due to technical limitations of the DHM related to the maximum measurable sample slope angle.
